# Supplementary material for: Hybrid Models and Biological Model Reduction with PyDSTool
Source: PLoS Comput Biol. 2012 Aug 9;8(8):e1002628. doi: 10.1371/journal.pcbi.1002628 (PMC3415397; doi:10.1371/journal.pcbi.1002628)
Supplement: Text S4 — Complete source code for the PyDSTool package (version 0.88.120504). Includes API documentation and help files linking to web pages. This file is identical to the current public release on Sourceforge.net. (ZIP) [file pcbi.1002628.s004.zip › PyDSTool/html/PyDSTool.Toolbox.phaseplane.inflection_zone_leaf-class.html]

xml version="1.0" encoding="ascii"?


PyDSTool.Toolbox.phaseplane.inflection\_zone\_leaf


| Home | Trees | Indices | Help | PyDSTool |
| --- | --- | --- | --- | --- |

Package PyDSTool ::
Package Toolbox ::
Module phaseplane ::
Class inflection\_zone\_leaf

[hide private]

[frames] | no frames]

# Class inflection\_zone\_leaf

source code

```
       object --+                    
                |                    
 MProject.feature --+                
                    |                
MProject.feature_leaf --+            
                        |            
 MProject.ql_feature_leaf --+        
                            |        
                    zone_leaf --+    
                                |    
              nullcline_zone_leaf --+
                                    |
                                   inflection_zone_leaf
```

---

```
A single inflection point zone.

pars:
  xtol --> precision of zone center finding
  find_exact -> exact x value of inflection (not implemented yet)
  zone_width -> positive scalar (default Inf grows to next inflection point)
```


|  |  |
| --- | --- |
| Instance Methods | [hide private] |

|  |  |
| --- | --- |
| evaluate(self, nullc) | source code |

**Inherited from `nullcline_zone_leaf`** (private):
`_grow_zone_by_radius`,
`_grow_zone_by_width`,
`_prepare`,
`_set_mutex_zone_by_width`

**Inherited from `zone_leaf`**:
`finish`

**Inherited from `MProject.feature_leaf`**:
`__repr__`,
`__str__`,
`set_ref_traj`

**Inherited from `MProject.feature_leaf`** (private):
`_residual_info`

**Inherited from `MProject.feature`**:
`__call__`,
`__eq__`,
`__hash__`,
`__init__`,
`__ne__`,
`info`,
`postprocess_ref_traj`,
`prepare`,
`reset_metric`,
`validate`

**Inherited from `MProject.feature`** (private):
`_find_idx`

**Inherited from `object`**:
`__delattr__`,
`__getattribute__`,
`__new__`,
`__reduce__`,
`__reduce_ex__`,
`__setattr__`


|  |  |
| --- | --- |
| Properties | [hide private] |

**Inherited from `object`**:
`__class__`


|  |  |
| --- | --- |
| Method Details | [hide private] |

|  |  |
| --- | --- |
| evaluate(self, nullc) | source code |

Overrides: MProject.feature.evaluate

  


| Home | Trees | Indices | Help | PyDSTool |
| --- | --- | --- | --- | --- |

|  |  |
| --- | --- |
| Generated by Epydoc 3.0.1 on Fri May 4 15:24:09 2012 | http://epydoc.sourceforge.net |
